# Increases in apoptosis, caspase activity and expression of *p53* and *bax*, and the transition between two types of mitochondrion-rich cells, in the gills of the climbing perch, *Anabas testudineus*, during a progressive acclimation from freshwater to seawater

**DOI:** 10.3389/fphys.2013.00135

**Published:** 2013-06-07

**Authors:** Biyun Ching, Xiu L. Chen, Jing H. A. Yong, Jonathan M. Wilson, Kum C. Hiong, Eugene W. L. Sim, Wai P. Wong, Siew H. Lam, Shit F. Chew, Yuen K. Ip

**Affiliations:** ^1^Department of Biological Science, National University of SingaporeKent Ridge, Singapore, Singapore; ^2^Ecofisiologia CIMARPorto, Portugal; ^3^NUS Environmental Research Institute, National University of SingaporeKent Ridge, Singapore, Singapore; ^4^Natural Sciences and Science Education, National Institute of Education, Nanyang Technological UniversitySingapore, Singapore

**Keywords:** air-breathing fish, Na^+^/K^+^-ATPase, Na^+^:K^+^:2Cl^−^ cotransporter, osmoregulation, seawater adaptation, TUNEL

## Abstract

This study aimed to test the hypothesis that branchial osmoregulatory acclimation involved increased apoptosis and replacement of mitochdonrion-rich cells (MRCs) in the climbing perch, *Anabas testudineus*, during a progressive acclimation from freshwater to seawater. A significant increase in branchial caspase-3/-7 activity was observed on day 4 (salinity 20), and an extensive TUNEL-positive apoptosis was detected on day 5 (salinity 25), indicating salinity-induced apoptosis had occurred. This was further supported by an up-regulation of branchial mRNA expression of *p53*, a key regulator of cell cycle arrest and apoptosis, between day 2 (salinity 10) and day 6 (seawater), and an increase in branchial p53 protein abundance on day 6. Seawater acclimation apparently activated both the extrinsic and intrinsic pathways, as reflected by significant increases in branchial caspase-8 and caspase-9 activities. The involvement of the intrinsic pathway was confirmed by the significant increase in branchial mRNA expression of *bax* between day 4 (salinity 20) and day 6 (seawater). Western blotting results revealed the presence of a freshwater Na^+^/K^+^-ATPase (Nka) α-isoform, Nka α1a, and a seawater isoform, Nka α1b, the protein abundance of which decreased and increased, respectively, during seawater acclimation. Immunofluorescence microscopy revealed the presence of two types of MRCs distinctly different in sizes, and confirmed that the reduction in Nka α1a expression, and the prominent increases in expression of Nka α1b, Na^+^:K^+^:2Cl^−^ cotransporter 1, and cystic fibrosis transmembrane conductance regulator Cl^−^ channel coincided with the salinity-induced apoptotic event. Since modulation of existing MRCs alone could not have led to extensive salinity-induced apoptosis, it is probable that some, if not all, freshwater-type MRCs could have been removed through increased apoptosis and subsequently replaced by seawater-type MRCs in the gills of *A. testudineus* during seawater acclimation.

## Introduction

The climbing perch, *Anabas testudineus* (Bloch), which belongs to Family Anabantidae and Order Perciformes, is commonly regarded as a freshwater teleost. It can be found in canals, lakes, ponds, swamps and estuaries in tropical Asia, and can tolerate extremely unfavorable water conditions (Pethiyagoda, 1991). It is an obligatory air-breather with accessory breathing organs (the labyrinth organs), which facilitate the utilization of atmospheric air, in the upper part of its gill-chambers (Munshi et al., 1986; Graham, 1997). During drought, *A. testudineus* stays in pools associated with submerged woods and shrubs (Sokheng et al., 1999) or buries under the mud (Rahman, 1989). It can also travel long distances on land between pools of water, covering several hundred meters per trip when the air is sufficiently humid (Davenport and Abdul Martin, 1990). On land, it can use amino acids as an energy source to support locomotor activity and actively excrete ammonia through its gills and skin (Tay et al., 2006). In addition, *A. testudineus* can acclimate from freshwater to seawater through a progressive increase in salinity in the laboratory (Chang et al., 2007).

Recently, Ip et al. (2012b) cloned and sequenced *cystic fibrosis transmembrane conductance regulator* (*cftr*) from the gills of *A. testudineus*. They reported that the branchial mRNA expression of *cftr* was low in fish kept in freshwater, but it increased significantly in fish acclimated to seawater for 1 day or 6 days, indicating that Cl^−^ excretion through the apical Cftr of the branchial epithelium was essential to seawater acclimation (Ip et al., 2012b). In addition, Loong et al. (2012) obtained a complete coding cDNA sequence of *Na*^+^:*K*^+^:2*Cl*^−^ *cotransporter 1* (*nkcc1*) from the gills of *A. testudineus* and reported that its mRNA expression and protein abundance increased significantly in the gills of fish acclimated to seawater. Hence, similar to marine teleosts that have a branchial transepithelial potential of ~25 mV (blood side positive), Cl^−^ enters the mitochondrion-rich cells (MRCs) through the basolateral Nkcc1 and is subsequently excreted through the apical Cftr in the gills of *A. testudineus*. Since the upregulation of Nkcc1 would lead to an increase in the intracellular Na^+^ concentration, a corresponding upregulation of basolateral Na^+^/K^+^-ATPase (Nka) activity would be necessary to remove the excess Na^+^ that had entered the MRCs. Indeed, Ip et al. (Ip et al., 2012a) identified three *nka* α-subunit isoforms, *nka* α*1a*, *nka* α*1b*, and *nka* α*1c*, from the gills of *A. testudineus*. The mRNA expression of *nka* α*1a* was down-regulated in the gills of fish acclimated to seawater, indicating that it was involved in branchial Na^+^ absorption in a hypoosmotic environment. By contrast, seawater acclimation led to an upregulation of the mRNA expression of *nka* α*1b* and to a lesser extent *nka* α*1c*, indicating that they were essential for ion secretion in a hyperosmotic environment.

Changes in MRC types are critical to branchial osmoregulatory acclimation in euryhaline freshwater fishes (Hiroi et al., 1999), which can be achieved through modulation of existing MRCs, especially when confronted with acute salinity changes. To date, studies on MRC modification for salinity acclimation have mostly been done on tilapia (*Oreochromis mossambicus*). Using 5-bromo-2′-deoxyuridine as a DNA marker, Tsai and Hwang (1998) observed the development of wheat germ agglutinin (WGA)-positive MRCs in the gills of tilapia during the first 2–4 day post-labeling, which then transformed into WGA-negative MRCs 6–8 days later, indicating the transition between different types (or subtypes) of MRCs. Hiroi et al. (1999) applied DASPEI vital staining and confocal microscopy to trace the sequential changes *in vivo* in individual MRCs in the skin of tilapia embryo transferred from freshwater to seawater. They demonstrated that most of the small freshwater-type MRCs could survive the osmotic shock and transformed into large seawater-type MRCs. They also reported that both types of MRCs were renewed from the presumably undifferentiated cells at similar turnover rates (Hiroi et al., 1999). Lin and Hwang (2004) used the same approach to demonstrate that >90% of the original MRCs in the yolk-sac membrane of tilapia survived, and modulated their apical membrane to enhance their Cl^−^ uptake capacity, after being transferred from water containing high concentrations of Cl^−^ to water containing low concentrations of Cl^−^. They reported that less than 10% of the MRCs were newly recruited.

Recently, using the scanning ion-selective electrode technique, Shen et al. (2011) demonstrated that individual MRCs could change functionally from ion uptake to ion secretion in the skin of medaka larvae during acute salinity change. Katoh and Kaneko (2003) also reported that seawater-type MRCs were transformed morphologically and functionally into freshwater-type cells as a short-term response when *Fundulus heteroclitus* was transferred directly from seawater to freshwater, but MRC replacement occurred subsequently as a long-term response. By contrast, Kammerer and Kültz (2009) and Inokuchi and Kaneko (2012) reported that branchial apoptosis occurred in *O. mossambicus* 6–24 h after being acutely transferred from freshwater to brackish water (salinity 25). They concluded that an acute transfer from hypoosmotic to hyperosmotic media enhanced the apoptosis of freshwater-type MRCs, leading to a reduction in the hyperosmoregulatory ability. It also stimulated the proliferation of seawater-type MRCs leading to an increase in the ability for hypoosmotic hypoionic regulation. Hence, there is an apparent controversy over whether MRC replacement involving apoptosis and cell proliferation is an adaptive response to acute or progressive/long-term salinity changes. Furthermore, it is unclear whether salinity-induced apoptosis of MRCs in fish gills is mediated via the extrinsic or the intrinsic pathway.

Apoptosis is important for differentiation of tissues and organ systems during development and for removing terminally damaged cells (Jones, 2001). It is regulated by a variety of cellular signaling pathways (Edinger and Thompson, 2004) and biochemical events, which lead to a series of cellular changes including blebbing, shrinkage, nuclear fragmentation, chromatin condensation and chromosomal DNA fragmentation, and result in cell death (Jones, 2001; Norbury and Hickson, 2001; Edinger and Thompson, 2004; Elmore, 2007). Known for its tumor suppressor role, p53 is a key regulator eliciting cellular response to a variety of stress signals (Riley et al., 2008; Vazquez et al., 2008). The activation of p53 can lead to apoptosis or cell-cycle arrest, and the final outcome is determined by the level of *p53* (Riley et al., 2008). A low p53 level favors cell-cycle arrest, while a higher p53 level triggers apoptosis (Laptenko and Prives, 2006). Functional conservation relative to mammalian p53 has been reported for zebrafish (see Storer and Zon, 2010 for a review). Similar to mammalian p53, zebrafish p53 is a transcriptional regulator of several of the same genes as mammals (Langheinrich et al., 2002; Lee et al., 2008), is necessary for apoptosis in response to DNA damaging agents (Langheinrich et al., 2002; Berghmans et al., 2005), and is negatively regulated by Murine Double Minute 2 (MDM2) (Langheinrich et al., 2002).

Apotosis is mainly orchestrated by an evolutionarily conserved family of intracellular cysteinyl aspartate-specific proteases known as caspases, which can be grouped into two categories: (1) upstream initiator caspases (caspase-2, -8, -9, and -10) and (2) downstream executioner or effector caspases (caspase-3, -6, and -7), (Meergans et al., 2000; Denault and Salvesan, 2003). The effector caspases are activated by upstream caspases, and they perform downstream execution steps of apoptosis by cleaving multiple cellular substrates. There are two main apoptosis signaling pathways. The extrinsic/death-receptor pathway is initiated through the tumor necrosis factor (TNF) family receptors (Salvesen and Dixit, 1997; Yuan, 1997) and/or dependency receptors (Mehlen and Bredesen, 2004), and involves the activation of procaspase-8. On the other hand, the intrinsic pathway of apoptosis involves the activation of procaspase-9 (Alberts et al., 2008), and occurs in the mitochondria which sequester a range of pro-apoptotic proteins (Hengartner, 2000). A highly regulated step within the intrinsic pathway of apoptosis is the release of cytochrome C which is controlled by a family of proteins regulating cytochrome c release (Taylor et al., 2008). These proteins are either pro-apoptotic such as Bcl-2-associated X protein (BAX) and Bcl-2-associated death promoter (BAD) or anti-apoptotic like B-cell lymphoma 2 (BCL2) and B-cell lymphoma extra-large (BCL-X_L_) (Norbury and Zhivotovsky, 2004; Roos and Kaina, 2006).

Therefore, the objective of this study was to test the hypothesis that increased apoptosis occurred in the gills of *A. testudineus* during a progressive acclimation from freshwater to seawater (salinity 30). TUNEL assay was used to detect apoptotic cells and to demonstrate that increased apoptosis occurred during branchial osmoregulatory acclimation in *A. testudineus* in brackish water, probably between salinity 10 and salintiy 25. To verify that salinity acclimation had induced apoptosis, efforts were made to determine the branchial activity of downstream executioner caspases-3/-7. In addition, we determined the branchial activities of upstream initiator caspases-8 and -9, to elucidate whether the intrinsic pathway and/or the extrinsic pathway were activated to initiate salinity-induced apoptosis. We also obtained full cDNA sequences of *p53* and *bax* from the gills of *A. testudineus*, with the aim of examining the effects of seawater acclimation on their branchial mRNA expression. Subsequently, Western blotting was performed, using custom-made antibodies, to examine the protein abundance of Nka α1a and Nka α1b in the gills of *A. testudineus* exposed to freshwater or seawater. Finally, immunofluorescence microscopy was performed to confirm that a transition between freshwater-type MRCs and seawater-type MRCs had indeed occurred during seawater acclimation, and to examine the time-course relationship between the changes in expression of Nka α1a, Nka α1b, Nkcc, and Cftr and salinity-induced apoptotic. The objective was to verify that a reduction in Nka α1a expression occurred before, and increases in expression of Nka α1b, Nkcc and Cftr occurred after, the peak of apoptosis in the gills of *A. testudineus*.

## Materials and methods

### Animals

Specimens of *A. testudineus* (25–45 g body mass) were purchased from a local fish distributor. Fish were kept in dechlorinated tap water (freshwater) at 25°C in fiber glass tanks with a continuous flow through system for at least 2 weeks under a 12 h light: 12 h dark regime before experiments. No aeration was provided because *A. testudineus* is an obligatory air-breather. They were fed frozen bloodworm once every two days. Approval to undertake this study was obtained from the Institutional Animal Care and Use Committee of the National University of Singapore (IACUC 021/10 and 098/10).

### Experimental conditions

In general, fish (*N* = 5) were exposed to daily increases in salinity from freshwater (day 1; control) to salinity 10 (day 2), salinity 15 (day 3), salinity 20 (day 4), salinity 25 (day 5), and salinity 30 (seawater; day 6), and kept in seawater for another 5 days until day 11. Saline waters were prepared by mixing freshwater with natural seawater in different proportions. Both freshwater and experimental fish were fed with frozen bloodworms. Feed was withdrawn two days prior to sample collection. Fish were killed with an overdose of neutralized 0.2% MS-222 (Sigma -Aldrich) followed with a strong blow to the head. Gills were quickly excised for fluorescence microscopy or TUNEL assay, or cooled in liquid N_2_ and stored at −80°C for or molecular work. Gill filaments excised from all four gill arches constituted one sample. Samples for caspase activity determination were collected separately from another set of experimental fish (*N* = 5 for each condition).

### Custom-made anti-Nka α1a and anti-Nka α1b monoclonal antibodies

We developed two custom-made isoform-specific monoclonal antibodies against Nka α1a (Genbank: AFK29492) and Nka α1b (Genbank: AFK29493) of *A. testudineus* (Ip et al., 2012a) through Abmart, Inc. (Shanghai, China). Epitopes (Nka α1a: KGKKNKDFDN; Nka α1b: LSLEEINRKY) that provide both high specificity and adequate immunogenicity were selected from the amino terminal regions for monoclonal antibody production. The two synthetic peptides were over-expressed in *E. coli* and purified by Ni-affinity chromatography before immunization. Three BALB/C mice per antigen were immunized and sacrificed after 24 days. Spleen cells were obtained and fused with SP2/0 myeloma cells for hybridomas generation (Abmart, Inc.). Clones were grown from single parent cells on microtitre wells in a selection medium containing hypoxanthine, aminopterin and thymidine. The antibodies secreted by the different clones were tested by ELISA (Abmart, Inc.). Stable clones with high ELISA titer were injected into the peritoneal cavity of mice to produce antibody-rich ascites fluids. After 14 days, the ascites fluids were collected and validated by western blot analysis using the mini-PROTEAN® II multiscreen apparatus (BioRad, Hercules, CA, USA). Gill samples of *A. testudineus* that were acclimatized to either freshwater or seawater were used as test antigens. The Nka α1a antibody detected a distinct band at ~110 kDa in freshwater condition but remained undetectable in the seawater condition. Similarly, the Nka α1b antibody detected a single band at ~110 kDa in the seawater condition but none in the freshwater condition. The molecular masses of the bands detected by the Nka α1a and Nka α1b antibodies were similar to the estimated molecular weight of *A. testudineus* Nka α1a and Nka α1b as reported by Ip et al. (2012a). Thus, the two Nka monoclonal antibodies were confirmed to be isoform-specific and no cross-reactivity was detected. The ascites fluids were then purified by Protein G affinity chromatography before they were used for immunohistochemistry.

### Sample preparation for tunel and immunofluorescence microscopy

Gills from three different fish (*N* = 3) for each of the four conditions, (1) freshwater on day 1, (2) salinity 15 on day 3, (3) salinity 25 on day 5, and (4) seawater (salinity 30) on day 6, during a progressive acclimation from freshwater to seawater, were excised. The freshly-excised samples were immersion fixed overnight in 3% paraformaldehyde in phosphate-buffered saline (PBS; pH 7.4) at 4°C. Samples were decalcified (FASC: 30% formic acid/ 13% sodium citrate, pH 2.3) at room temperature for 24 h, dehydrated in ethanol and cleared in xylene before embedding in paraffin. Paraffin sections (5 μm) were collected onto 3-aminopropyltriethoxysilane (Sigma-Aldrich Co., St. Louis, MO, USA) coated slides, and used for TUNEL assay and immunofluorescence microscopy work.

### Tunel assay

DNA fragmentation within apoptotic cells in the gill filaments of the first and second gill arches from three different fish for each of the four conditions were determined using TACS® 2 TdT-Fluor *in situ* apoptosis detection kit (Trevigen, Gaithersburg, MD, USA), according to the manufacturer's protocol. Briefly, sections were pretreated with 20 μg/ml of proteinase K for 15 min and equilibrated in 1x TdT labeling buffer for 5 min at room temperature. Each sample was subsequently incubated with 50 μl of labeling reaction mix containing Co^2+^ as the signal enhancing cation for 1 h at 37°C. The reaction was terminated with 1x TdT stop buffer and sections labeled with Streptavidin-Alexa Fluor® 488 (Life Technologies Inc.). Sections were also counterstained with nuclear stain DAPI.

Sections were viewed on a Leica DM 6000B epifluorescence microscope (Leica Microsystems, Mannheim, Germany) and images captured using a Leica DFC 340 FX digital camera. Optimal exposure settings were predetermined and all images captured under these settings. Brightness and contrast of the plates were adjusted while maintaining the integrity of the data.

### Immunofluorescence microscopy

Immunofluorescence microscopy was performed on gill filaments of the first and second gill arches from three different fish for each of the four conditions. Antigen retrieval was performed by treating deparaffinized sections with 0.05% citraconic anhydride (Namimatsu et al., 2005). Sections were subsequently labeled using (1) anti-Nka α1a or Nka α1b custom-made mouse monoclonal antibodies (Abmart Inc., Shanghai, China), (2) anti-NKCC/NCC mouse monoclonal antibody (T4; Developmental Studies Hybridoma Bank, Iowa City, IA, USA), or (3) anti-CFTR mouse monoclonal antibody (Clone #24-1; R&D systems, Minneapolis, MN, USA). They were double labeled with (4) NKA αRb1 rabbit polyclonal antibody, which is a pan-specific antibody originally designed by Ura et al. (1996) for labeling Nka α-subunit isoforms and widely used for fish species (Wilson, 2007). Antibodies (1) and (2) were diluted 1:100 in blocking buffer (1% BSA in TPBS); antibody (3) was diluted 1:100 using the HIKARI signal enhancement solution A (Nacalai Tesque Inc., Kyoto, Japan); and, antibody (4) was diluted 1:500 in 1% BSA in TPBS. Primary antibody incubations were performed overnight at 4°C. Secondary antibody incubations using goat anti-mouse Alexa Fluor® 568 and goat anti-rabbit Alexa Fluor® 488 (1:500 dilution; Life Technologies Inc.) were carried out at 37°C for 1 h. After primary and secondary antibody incubations, sections were rinsed three times with TPBS and mounted. Slides were viewed and images captured as described for the TUNEL assay. The corresponding differential interference contrast (DIC) image was also captured for tissue orientation.

### SDS-PAGE electrophoresis and western blotting of Nka α-subunit isoforms

The gill filaments were homogenized three times in five volumes (w/v) of ice cold buffer containing 50 mmol l^−1^ Tris HCl, (pH 7.4), 1 mmol l^−1^ EDTA, 150 mmol l^−1^ NaCl, 1 mmol l^−1^ NaF, 1 mmol l^−1^ Na_3_VO_4_, 1% Nonidet P-40, 1% sodium deoxycholate, 1 mmol l^−1^ Phenylmethylsulfonyl fluoride, and 1× HALT protease inhibitor cocktail (Thermo Fisher Scientific, Rockford, IL, USA) at 24,000 rpm for 20 s each with 10 s intervals using the Polytron PT 1300D homogenizer (Kinematica AG, Lucerne, Switzerland). The homogenate was centrifuged at 10,000 × g for 20 min at 4°C. The protein concentration in the supernatant obtained was determined as mentioned previously, and adjusted to 2 μg μl^−1^ with Laemmli buffer (Laemmli, 1970). Samples were heated at 70°C for 15 min, and then kept at −80°C until analysis.

Proteins were separated by SDS-PAGE (8% acrylamide for resolving gel, 4% acrylamide for stacking gel) under conditions as described by Laemmli (1970) using a vertical mini-slab apparatus (Bio-Rad). They were then electrophoretically transferred onto PVDF membranes using a transfer apparatus (Bio-Rad). After transfer, membranes were blocked with 10% skim milk in TTBS (0.05% Tween 20 in Tris-buffered saline: 20 mmol l^−1^ Tris-HCl; 500 mmol l^−1^ NaCl, pH 7.6) for 1 h before being incubated overnight at 4°C with anti-Nka α1a or anti-Nka α1b custom-made monoclonal antibodies (Abmart, Inc.) or anti-NKA (α5; Developmental Studies Hybridoma Bank, Iowa City, IA, USA) (1:800 dilution), or pan-actin antibody (1:5000 dilution; DSHB). The anti-NKA α5 antibody was developed by Douglas M. Farmbrough (Johns Hopkins University, MD, USA) and is known to react pan-specifically with Nka α-subunit isoforms in fish and other animals. All primary antibodies were diluted in 1% BSA in TTBS. The membranes were then incubated in alkaline phosphatase-conjugated secondary antibodies (anti-mouse; 1:10,000 dilution) for 1 h, rinsed and then incubated for 5 min in a solution of 5-bromo-4-chloro-3-indolyl phosphate p-toluidine salt and nitro-blue tetrazolium chloride (Life Technologies Inc., Carlsbad, CA, USA) for color development. Color method was adopted because of the large linearity range which could accommodate the large differences in protein abundance of Nka α-isoforms between gills samples of fish exposed to various experimental conditions. The blots were scanned using CanonScan 4400F flatbed scanner in TIFF format at 300 dpi resolution. Densitometric quantification of band intensities were performed using ImageJ (version 1.40, NIH), calibrated with a calibrated 37 step reflection scanner scale (1″ × 8″; Stouffer #R3705-1C).

### Sample collection for and the determination of caspase activities

Fish (*N* = 5) were killed on day 1 (freshwater; control), day 2 (1 day in salinity 10), day 3 (1 day in salinity 15), day 5 (1 day in salinity 25), day 6 (1 day in seawater) and day 11 (6 days in seawater). Another group of fish (*N* = 5) kept in freshwater was killed on day 11 to serve as another control. Gill filaments excised from all four gill arches constituted one sample. They were frozen in liquid nitrogen and stored at −80°C until analysis which were performed within one month of sampling. Frozen gill samples (*N* = 5) were weighed and ground to powders in liquid nitrogen before 20 volumes (w/v) of ice cold homogenizing buffer at pH 7.2, containing 20 mmol l^−1^ KCl; 20 mmol l^−1^ Hepes; 2 mmol l^−1^ MgCl_2_; 1 mmol l^−1^ EDTA; 4 mmol l^−1^ Dithiothreitol; 1:1000 of HALT™ proteases inhibitor cocktail kit (Pierce Biotechnology, Inc., IL, USA) was added to each sample. The mixture was homogenized three times for 20 s each with 10 s intervals using an Ika-werk Staufen Ultra-Turrax homogenizer (Janke and Kundel, Stanfeni, Germany) at 24,000 rpm. The samples were then centrifuged for 30 min at 10,000 × g at 4°C. The supernatant obtained was diluted appropriately with homogenizing buffer for the determination of caspase activities.

Caspase-3/-7, caspase-8 and caspase-9 activities were determined using various caspase specific fluorometric assay kits purchased from Calbiochem Inc. (Darmstadt, Germany). The determination of the activity of each individual caspase enzyme was based on the hydrolytic cleavage of a specific peptide sequence with aspartate residues being labeled with the fluorescent molecule, 7-amino-4-trifluoromethyl coumarin, resulting in the release of the fluorophore and causing a blue to green shift in fluorescence. For the caspase assays, 50 μl of the diluted supernatant for each sample and 50 μl of assay buffer were pipetted into a 96-well plate. Specific substrate conjugate was added to the reaction mixture to start the reaction, which was recorded immediately using a multi-well fluorescence plate reader (Molecular Devices, CA, USA) at excitation/emission wavelengths of 400/505 nm at 28°C. The increase in the relative fluorescence units (RFU) were recorded as a kinetic plot over time. The caspase activity was expressed as RFU min^−1^ mg^−1^ protein. Sample protein concentrations were determined by the method of Bradford (1976) with a microplate spectrophotometer (Molecular Devices) at 595 nm.

### Total RNA extraction and cDNA synthesis

The total RNA of the gill sample was extracted using Tri ReagentTM (Sigma-Aldrich Co., St. Louis, MO, USA), and further purified using the Qiagen RNeasy Mini Kit (Qiagen GmbH, Hilden, Germany). Following isolation, RNA was quantified spectrophotometrically using a Hellma traycell (Hellma GmbH & Co. KG, Müllheim, Germany). The RNA quality was checked electrophoretically to verify RNA integrity and RNA was stored at −80°C. First strand cDNA was synthesized from 1 μg of total RNA using oligo(dT)_18_ primer and the RevertAid™ first stand cDNA synthesis kit (Fermentas International Inc., Burlington, ON, Canada).

### Polymerase chain reaction (PCR), cloning and rapid ampification of cDNA ends (race) for *p53* and *bax*

PCR was carried out to obtain partial sequences using DreamTaq™ polymerase (Fermentas International Inc.) and the primers described in Table 1. The cycling conditions were 94°C (3 min), followed by 40 cycles of 94°C (30 s), 55°C (30 s), 72°C (2 min), and a final extension at 72°C (10 min). PCR products were separated by agarose gel electrophoresis. Bands of the estimated size were excised and purified using FavorPrep™ Gel Purification Mini Kit (Favorgen Biotech Corp., Ping-Tung, Taiwan). The purified PCR products were subjected to cycle sequencing using BigDye® Terminator v3.1 Cycle Sequencing Kit (Applied Biosystems Inc., Foster City, CA, USA) and the 3130XL Genetic Analyzer (Applied Biosystems Inc.). The PCR products obtained were ligated into pGEM®-T easy vector (Promega Corporation, Madison, WI, USA). JM109 *Escherichia coli* competent cells were transformed using the ligated vector and plated on Luria-Bertani (LB) agar with ampicillin, isopropyl β-D-1-thiogalactopyranoside (IPTG) and bromo-chromo-iodolyl-galactopyranoside (X-gal). Colony-PCR was performed on selected white colonies. Colonies with insert of estimated size were grown overnight in LB/ampicillin broth in a shaking incubator (37°C, 250 rpm). Plasmid extraction was performed using AxyPrep™ Plasmid Miniprep Kit (Axygen Biosciences, Union City, CA, USA), and plasmid concentration was determined spectrophotometrically and sequenced. Gene-specific RACE primers (Table 1) were designed using the partial sequences obtained. Total RNA (1 μ g) was reverse transcribed into RACE-ready cDNA using SMARTer™ RACE cDNA Amplification kit (Clontech Laboratories Inc., Mountain View, CA, USA). RACE-PCR was carried out using Advantage® 2 PCR kit (Clontech Laboratories Inc.) with 30 cycles of 94°C (30 s), 68°C (30 s), and 72°C (4 min). RACE-PCR products were separated using gel electrophoresis, purified and sequenced. One round of sequencing was carried out with sequencing primers (Table 1) to extend the partial sequences for *bax*. The partial fragments were aligned using BioEdit version 7.0.9.0 (Hall, 1999) to obtain the coding region which was translated to obtain predicted amino acid sequences.

**Table 1:** **Primers for PCR or RACE PCR to amplify or quantitative RT-PCR to quantify mRNA expression of *p53* or *Bcl-2 associated X protein* (*bax*) from the gills of *Anabas testudineus***.

**Gene**	**Primer type**		**Primer sequence (5′ to 3′)**
*p53*	PCR	Forward	CTGAACAARCTSTWCTGCCAG
		Reverse	CRCACACRCGCACYTCAAA
	RACE-PCR	5′ RACE	GGGCTCATAAGGGACAGTCACACTCT
		3′ RACE	CGTGGCTGATGTGGTCCGTAGATGTCCC
	qPCR	Forward	TTACTCCCTCCATCTCCACC
		Reverse	TCATCAAACCCTTCAGCCAG
*bax*	PCR	Forward	GTTGCTCTGTTCTACTTTGC
		Reverse	GGCAGTGAGGACACCAGC
	RACE-PCR	5′ RACE	CCTTGCTCCCTGATCCAGTTTATCACA
		3′ RACE	GCTCTGTTCTACTTTGCCTGTCG
		Sequencing 3′ RACE	TGGACCATGGATTACCTC
	qPCR	Forward	ACTCCTCACTCAGTCCCAC
		Reverse	GCAAAGTAGAACAGAGCAACCA

### Quantitative real-time PCR (qPCR)

RNA (1 μg) was reverse transcribed using random hexamer primers with RevertAid™ first strand cDNA synthesis kit (MBI Fermentas Inc., Burlington, ON, Canada). Real-time PCR was performed in triplicates with forward and reverse primers (Table 1) and KAPA SYBR® FAST Master Mix (2X; Kapa Biosystems, Woburn, MA, USA) in a StepOnePlus™ Real-Time PCR System (Applied Biosystems Inc., Foster City, CA, USA). Cycling conditions were 95°C (20 s) followed by 50 cycles of 95°C (3 s) and 60°C of (30 s).

Absolute quantification of transcripts in the qPCR reaction was carried out following the methods of Loong et al. (2012) and Ip et al. (2012a,b). In order to determine the absolute quantity of *p53* and *bax* transcripts separately in a qPCR reaction, efforts were made to produce two pure amplicons of defined regions of *p53* and *bax* cDNA from the gills of *A. testudineus* through PCR with qPCR primers (Table 1). Standard curves were obtained from plotting threshold cycle (C_T_) on the Y-axis and the natural log of concentration (copies of transcripts μl^−1^) on the X-axis. The standard cDNA template was serially diluted (from 10^9^ to 10^2^ copies), and standard curve was obtained from plotting threshold cycle (C_T_) against the natural log of copy number on the X axis. The amplification efficiencies for *p53* and *bax* were 99.2% and 97.8%, respectively. The quantity of transcript in a sample was determined from the linear regression line derived from the standard curve and the copies of transcript per ng cDNA.

### SDS-PAGE and western blotting of p53

Western blotting using commercially available antibodies was performed to confirm that there were indeed changes in protein expression of p53 in the gills of *A. testudineus* exposed to salinity 20 on day 4, salinity 25 on day 5 and seawater on day 6 during a progressive acclimation from freshwater to seawater. Attempts were made to acquire a commercial antibody that would react with Bax from *A. testudineus*, but to no avail.

The gill filaments were homogenized and proteins were separated by SDS-PAGE as described above. Proteins were then electrophoretically transferred onto PVDF membranes using a transfer apparatus (Bio-Rad). After transfer, membranes were blocked with 10% skim milk in TTBS (0.05% Tween 20 in Tris-buffered saline: 20 mmol l^−1^ Tris-HCl; 500 mmol l^−1^ NaCl, pH 7.6) for 1 h before being incubated overnight at 4°C with anti-p53 antibody (dilution 1:1000; catalogue number BL1371, Bethyl Laboratories Inc., Montgomery, TX, USA). The membranes were then incubated in horseradish peroxidase-conjugated secondary antibodies (anti-rabbit, dilution 1:10,000; catalogue number SC-2004, Santa Cruz Biotechnology, CA, USA) for 1 h at 25°C. The bands were subsequently detected by enhanced chemiluminescence (Western Lightning, PerkinElmer Life Science, Inc., Boston, MA. USA) due to the relatively low protein abundance of p53. After exposure to X-ray film for an optimal period of time, densitometric quantification of band intensities were performed using ImageJ, calibrated with a calibrated 37 step reflection scanner scale (1″ × 8″; Stouffer #R3705-1C). Since the protein abundance of p53 was much lower than other commonly used house-keeping proteins (e.g., actin and tubulin), it would not be technically feasible to analyze them together using the same protein concentration and exposure period. Hence, results were expressed as arbitrary densitometric unit per 100 μg protein, with reference to the protein concentration.

#### Statistical analysis

Results on caspase activities, mRNA expression and western blotting are presented as means + standard error of the mean (S.E.M.). One-way analysis of variance (ANOVA) followed by the Tukey *post-hoc* test were used to evaluate differences between means. Differences with *P* < 0.05 were regarded as statistically significant. No statistical analysis was applied to TUNEL assay because no TUNEL positive apoptosis was detectable in the freshwater control.

## Results

### Tunel positive apoptosis

**Figure 1:**
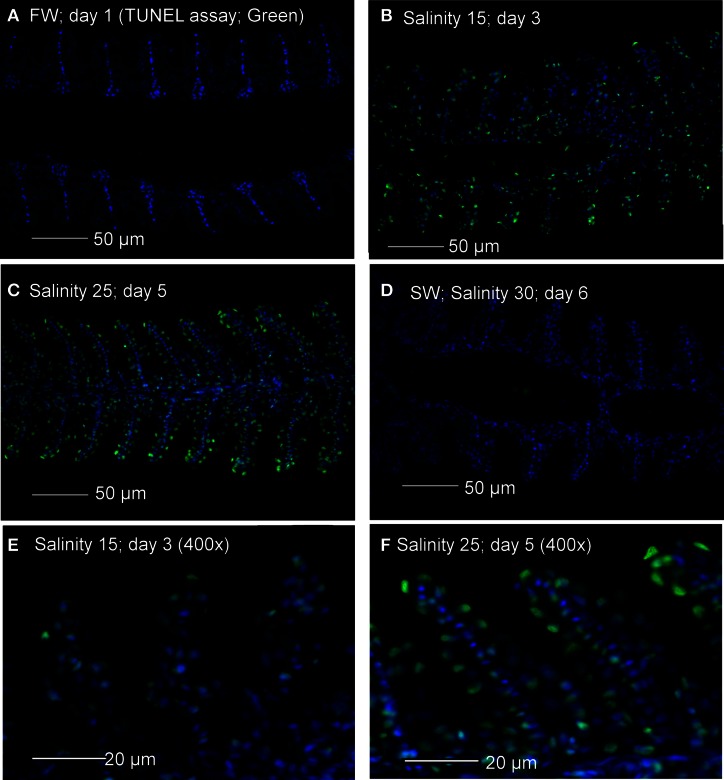
**Apoptotic cells indicated by TUNEL-positive nuclei (green) in gill sections of *Anabas testudineus* kept in (A) freshwater on day 1 (FW; control), or after 24 h of exposure to (B) salinity 15 on day 3, (C) salinity 25 on day 5 or (D) salinity 30 on day 6 after a progressive increase in salinity. All sections were counterstained with nuclear stain DAPI (blue).** Magnification: 200×. Reproducible results were obtained from three individuals for each of the four conditions. Micrographs for **(E)** salinity 15 and **(F)** salinity 25 were enlarged (Magnification: 400×) for clearer visualization of apoptotic nuclei.

TUNEL positive apoptosis was undetectable in gills of *A. testudineus* kept in freshwater, but was detected weakly and strongly in gills of fish exposed to salinity 15 (day 3) and salinity 25 (day 5), respectively, during a 6-day progressive acclimation from freshwater to seawater (Figure 1). By day 6, TUNEL positive apoptosis was no longer detectable in the gills of fish that had been exposed to seawater for 24 h (Figure 1).

### Activities of caspases

**Figure 2:**
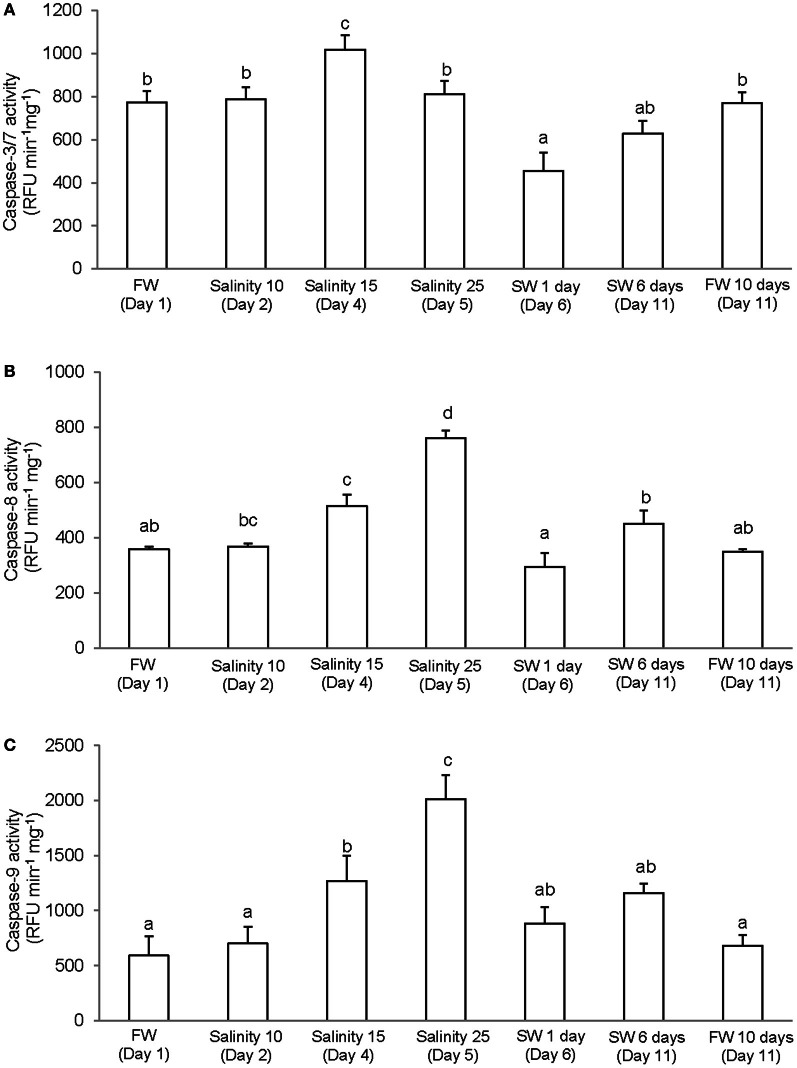
**Specific activity (RFU min^−1^ mg^−1^ protein) of (A) caspase-3/-7, (B) caspase-8, and (C) caspase-9 from the gills of *Anabas testudineus* kept in freshwater (FW; control) for 1 day or 11 days, or exposed to 6 days of progressive increase in salinity, that is day 2 in salinity 10, day 3 in salinity 15, day 5 in salinity 25, and day 6 in seawater (SW; salinity 30), followed by acclimation in seawater for another 5 days (day 11).** Values are means + S.E.M. (*N* = 4). Means not sharing the same letter are significantly different (*P* < 0.05).

It has been reported that, in some cases, commercially available caspase-specific substrates and inhibitors are highly promiscuous and results obtained may not indicate activities of the specific caspase (Chauvier et al., 2007; McStay et al., 2008). However, the differences in levels of specific activity and patterns of salinity-induced changes obtained from the gills of *A. testudineus* indicate that our results represent the activities of three different types of caspase. There were significant increase and decrease in activity of caspase-3/-7 in the gills of *A. testudineus* exposed to salinity 15 (day 3) and after 1 day of exposure to seawater on day 6, respectively (Figure 2A). In addition, a significant increase in branchial activity of caspase-8 occurred in fish exposed to salinity 25 (day 5; Figure 2B). As for caspase-9, there were significant increases in activity at salinity 15 (day 3) and salinity 25 (day 5) during a 6-day progressive acclimation from freshwater to seawater (Figure 2C).

### *p53/*p53

**Figure 3:**
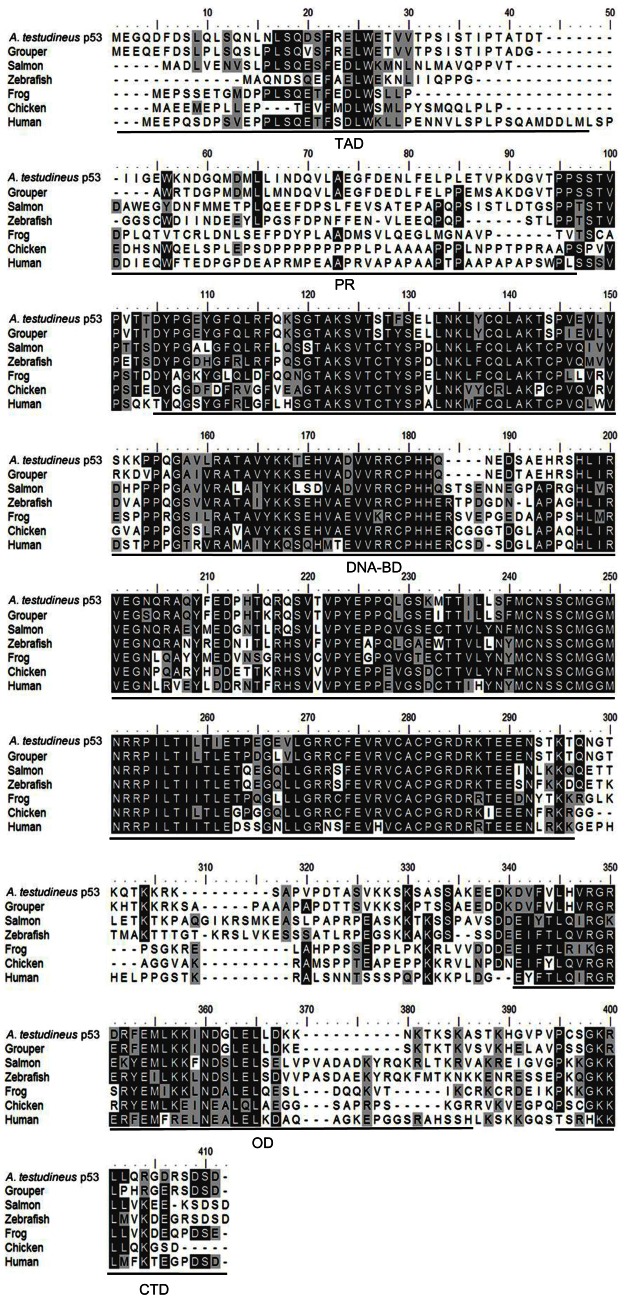
**A multiple alignment of the deduced amino acid sequence of p53 from gills of *Anabas testudineus* (Genbank accession number KC513732) with those from *Epinephelus coioides* (grouper; ADN04912.1), *Oreochromis niloticus* (tilapia; ADE21938.1), *Xiphophorus maculatus* (platyfish; AAC31134.1), *Salmo salar* (salmon; ACN10490.1), *Ictalurus punctatus* (catfish; NP_001187005.1), *Danio rerio* (zebrafish; NP_571402.1), *Xenopus laevis* (frog; CAA54672.1), *Gallus gallus* (chicken; NP_990595.1), *Mus musculus* (mouse; BAA82344.1), and *Homo sapiens* (human; BAC16799.1).** Identical amino acids are indicated by shaded black residues and similar amino acids (threshold value 50%) are indicated by shaded grey residues. Domains present, as indicated by line, are transactivation domain (TAD), proline-rich region (PR), DNA binding domain (DNA-BD), oligomerization domain (OD) and carboxy-terminal regulatory domain (CTD). Important residues are in boxes. Within the TAD, key residues are F^19^, W^23^, and L^26^ which make crucial Murine Double Minute 2 (MDM2) contact, and S^15^, T^18^, and S^20^ which are important phosphorylation sites. Within the DNA-BD, key residues that make direct contact with DNA are K^120^, S^241^, R^248^, R^273^, A^276^, C^277^, and R^280^. Within the OD, there is conserved R^333^, G^334^, E^349^, and highly conserved intermolecular salt bridge between R^337^ and D^352^. L^348^ has also been found to be one of the key hydrophobic residues in this region, while L^344^ is part of a leucine-rich nuclear export signal. In the CTD, K^381^, K^382^, K^386^, and S^392^ are important regions for posttranslational modification.

**Figure 4:**
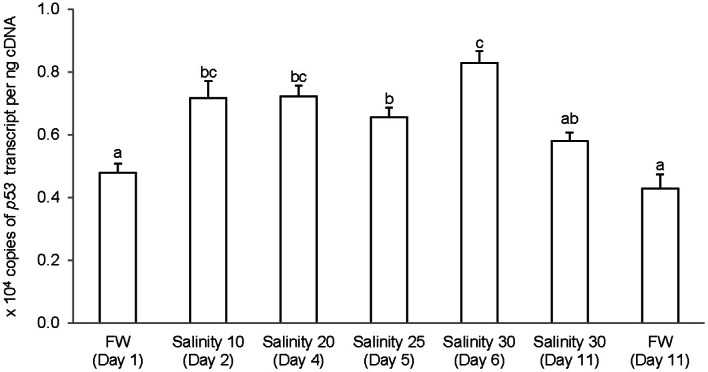
**Absolute quantification of *p53* mRNA (copies of transcript per ng cDNA) in the gills of *Anabas testudineus* kept in freshwater (FW) for 1 or 11 days (controls), or exposed to a 5-day progressive increase in salinity (S) from FW (day 1) to S 10 on day 2, S 20 on day 4, S 25 on day 5 and S 30 (seawater) on day 6, and then kept in seawater for another 6 days (day 11).** Results are presented as means ± S.E.M (*N* = 5). Means not sharing the same letter are significantly different (*P* < 0.05).

**Figure 5:**
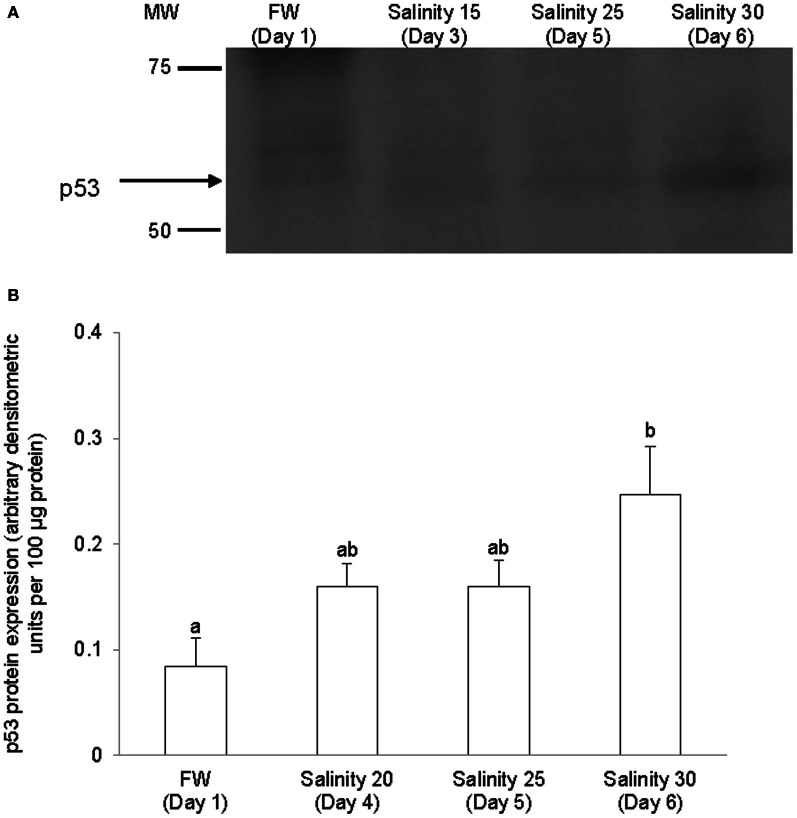
**Protein abundance of p53 in the gills of *Anabas testudineus* kept in freshwater (FW, control) or exposed to a 5-day progressive increase in salinity (S) from FW (day 1) to S 15 on day 3, S 25 on day 5 or S 30 (seawater) on day 6. (A)** Immunoblots of p53. **(B)** The protein abundance of p53 expressed as arbitrary densitometric units per 100 μg protein. Results represent mean + S.E.M. (*N* = 4). Means not sharing the same letter are significantly different (*P* < 0.05).

The complete coding cDNA sequence of *p53* (1143 bp) obtained from the gills of *A. testudineus* (Genbank accession number KC513732) putatively coded for 380 amino acids with an estimated molecular mass of 42.4 kDa (Figure 3). p53 of *A. testudineus* has a DNA-binding domain (DNA-BD) and a oligomerization domain flanked by intrinsically disordered regions at the C-terminal and N-terminal regions (Figure 3). The C-terminal region is a natively unfolded region consisting of regulatory domains while the N-terminal region comprises of an intrinsically disordered transactivation domain and a proline-rich region. The mRNA expression of *p53* in the gills of *A. testudineus* kept in freshwater for 1 day was comparable to that of fish kept in freshwater for 11 days (Figure 4). However, during seawater acclimation, there were significant increases in the mRNA expression of *p53* in the gills of fish exposed to salinity 10 (day 2), salinity 20 (day 4), salinity 25 (day 5), and salinity 30 (seawater; day 6; Figure 4). After 6 days of acclimation to seawater (day 11), the mRNA expression of *p53* in the gills returned to a level comparable to that of the day 1 freshwater control (Figure 4). There was a general trend of increase in the protein abundance of p53 in the gills of fish during seawater acclimation, and the branchial p53 protein abundance of fish exposed to seawater on day 6 was significantly greater than that of the freshwater control (Figure 5).

### bax

**Figure 6:**
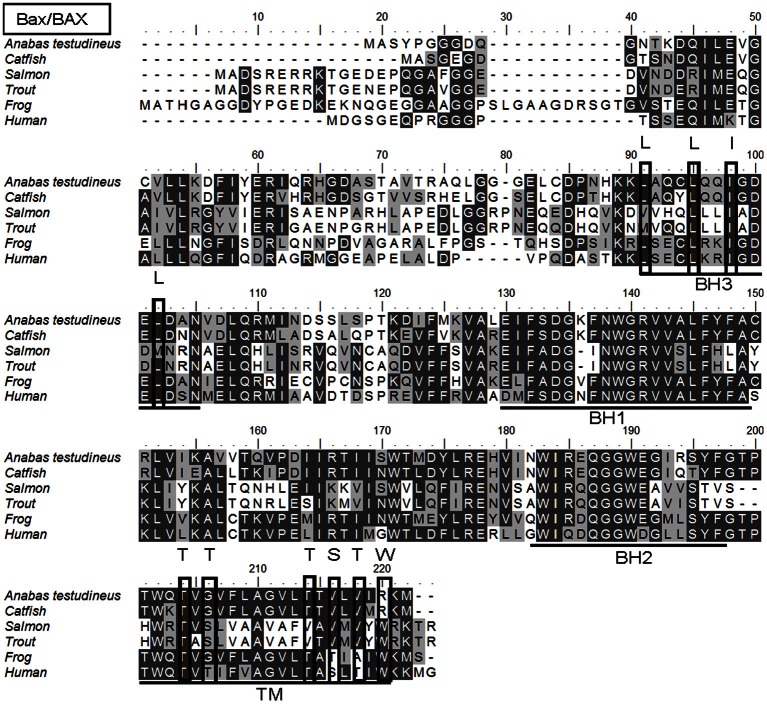
**A multiple alignment of the deduced amino acid sequence of Bcl-2 associated X protein from gills of *Anabas testudineus* (Genbank accession number KC513734) with those from *Homo sapiens* (human; NP_620116.1), *Mus musculus* (mouse; NP_031553.1), *Canis lupus familiaris* (chicken; NP_001003011.1), *Xenopus (Silurana) tropicalis* (frog; NP_989185.1), *Salmo salar* (salmon; ACI67445.2), *Oncorhynchus mykiss* (trout; ACO08752.1), and *Ictalurus punctatus* (catfish; NP_001187866.1).** Identical amino acids are indicated by shaded black residues and similar amino acids (threshold value 50%) are indicated by shaded grey residues. Domains and regions present, as indicated with lines, are Bcl-2 Homology 1 (BH1), Bcl-2 Homology 2 (BH2), Bcl-2 Homology 3 (BH3), and transmembrane region (TM). Important residues are in boxes. Within the BH3, important residues are L^59^, L^63^, I^66^, and L^70^ which are essential for binding to other BH3 proteins. In the TM, T^172^, T^174^, T^182^, S^184^, and T^186^ are amino acids containing hydroxyl groups, while S^184^ and W^188^ are important for Bax/BAX translocation from the cytosol to the mitochondria.

**Figure 7:**
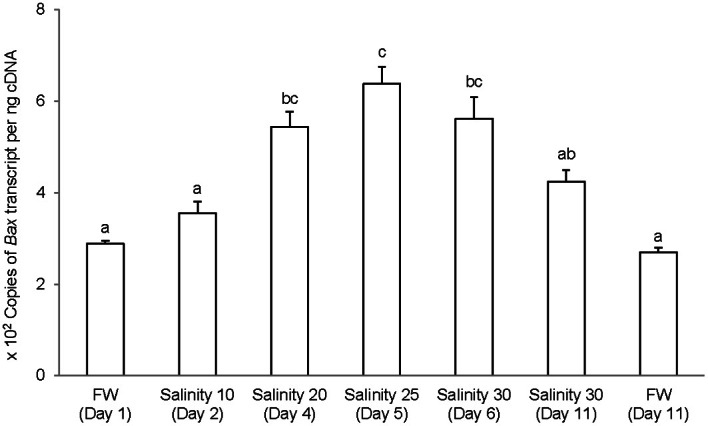
**Absolute quantification of *Bcl-2 associated X protein* (*bax*) mRNA (copies of transcript per ng cDNA) in the gills of *Anabas testudineus* kept in freshwater (FW) for 1 or 11 days (controls), or exposed to a 5-day progressive increase in salinity (S) from FW (day 1) to S 10 on day 2, S 20 on day 4, S 25 on day 5 and S 30 (seawater) on day 6, and then kept in seawater for another 6 days (day 11).** Results are presented as means ± S.E.M (*N* = 5). Means not sharing the same letter are significantly different (*P* < 0.05).

The complete coding cDNA sequence of *bax* obtained from the gills *A. testudineus* contained 579 bp (Genbank accession number KC513734). It putatively coded for 192 amino acids with an estimated molecular mass of 21.5 kDa (Figure 6). Bax of *A. testudineus* has Bcl-2 Homology 1, Bcl-2 Homology 2, Bcl-2 Homology 3 and transmembrane regions. The mRNA expression of *bax* in the gills of *A. testudineus* kept in freshwater for 1 day was comparable to that of fish kept in freshwater for 11 days (Figure 7). During seawater acclimation, there were significant increases in the mRNA expression of *bax* in the gills of fish exposed to salinity 20 (day 4), salinity 25 (day 5), and seawater (day 6; Figure 7). However, unlike *p53*, a significant increase in the mRNA expression of *bax* was not observed in the gills of fish exposed to salinity 10 (day 2). After 6 days of acclimation to seawater (day 11), the mRNA expression of *bax* in the gills of *A. testudineus* was comparable to that of the day 1 freshwater control (Figure 7).

### Western blotting of Nka α-subunit isoforms

**Figure 8:**
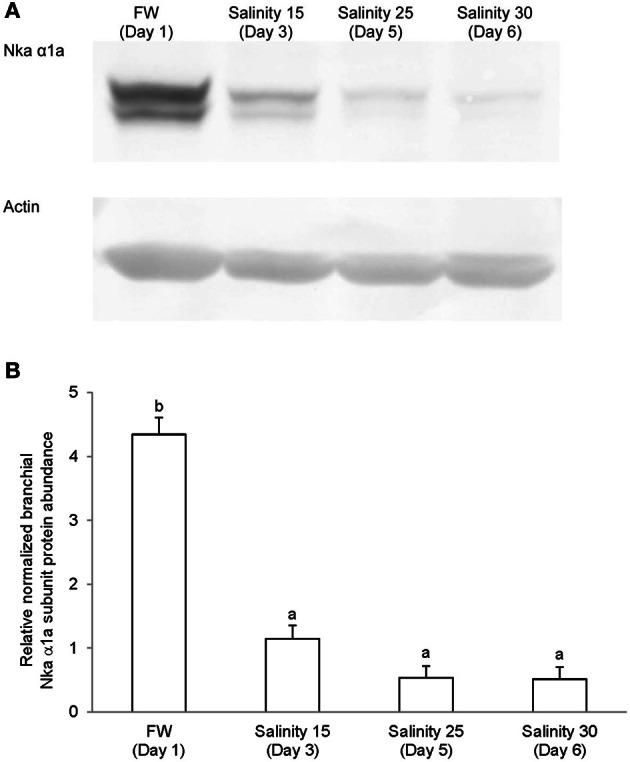
**Protein abundance of Na^+^/K^+^-ATPase α1a (Nka α1a) in the gills of *Anabas testudineus* kept in freshwater on day 1 (FW; control), or after 24 h of exposure to salinity 15 on day 3, salinity 25 on day 5, or salinity 30 (seawater) on day 6 during a progressive acclimation from freshwater to seawater. (A)** Immunoblots of Nka α 1a and actin. **(B)** The intensity of the Nka α 1a protein band normalized with respect to actin. Results represent mean + S.E.M. (*N* = 4). Means not sharing the same letter are significantly different (*P* < 0.05).

**Figure 9:**
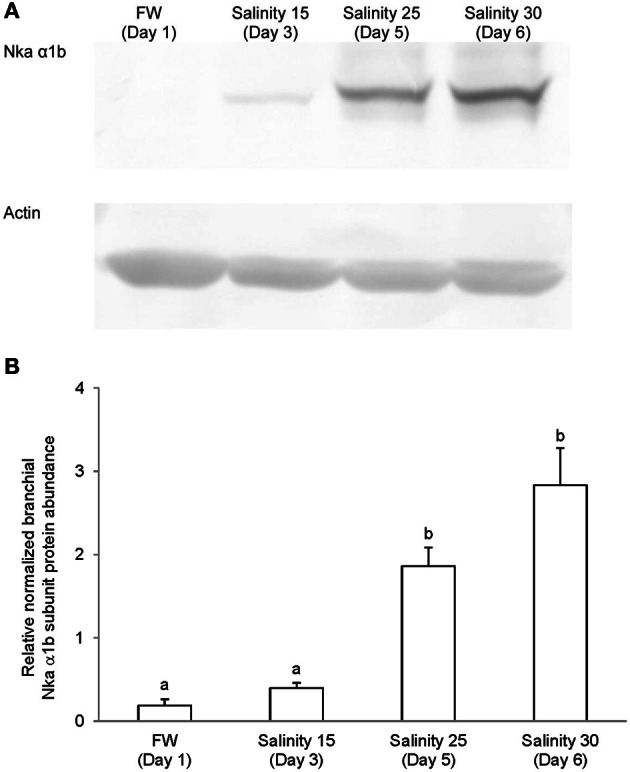
**Protein abundance of Na^+^/K^+^-ATPase α1b (Nka α1b) in the gills of *Anabas testudineus* kept in freshwater on day 1 (FW; control), or after 24 h of exposure to salinity 15 on day 3, salinity 25 on day 5, or salinity 30 (seawater) on day 6 during a progressive acclimation from freshwater to seawater. (A)** Immunoblots of Nka α 1b and actin. **(B)** The intensity of the Nka α 1b protein band normalized with respect to actin. Results represent mean + S.E.M. (*N* = 4). Means not sharing the same letter are significantly different (*P* < 0.05).

**Figure 10:**
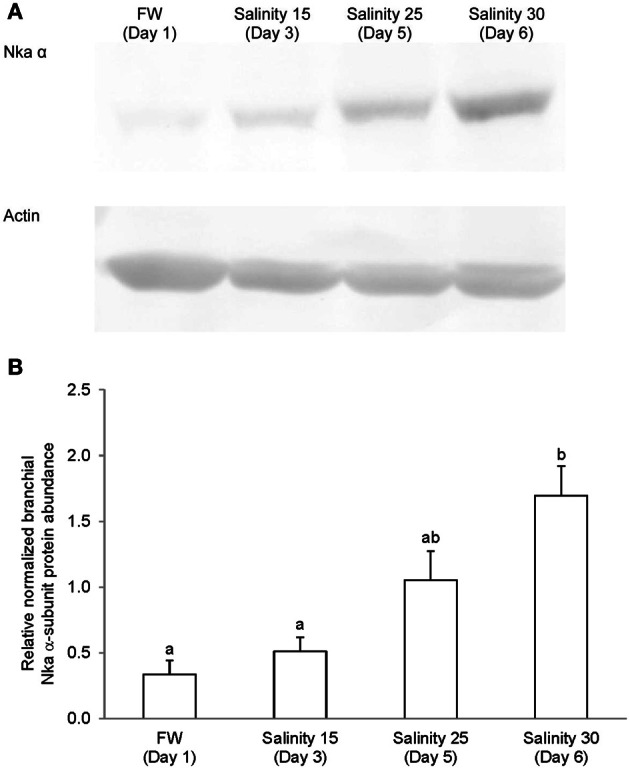
**Protein abundance of Na^+^/K^+^-ATPase (Nka) α-subunit in the gills of *Anabas testudineus* kept in freshwater on day 1 (FW; control), or after 24 h of exposure to salinity 15 on day 3, salinity 25 on day 5, or salinity 30 (seawater) on day 6 during a progressive acclimation from freshwater to seawater. (A)** Immunoblots of Nka α-subunit and actin. **(B)** The intensity of the Nka α-subunit band normalized with respect to actin. Results represent mean + S.E.M. (*N* = 4). Means not sharing the same letter are significantly different (*P* < 0.05).

Immunoblotting of gill samples from *A. testudineus* exposed to a progressive increase in salinity from freshwater to seawater using custom-made anti-Nka α1a or anti-Nka α1b monoclonal antibodies confirmed that Nka α 1a was a freshwater isoform while Nka α 1b was a seawater isoform. There was a significant decrease in the protein abundance of Nka α 1a in the gills of fish exposed to salinity 25 (day 5) and to seawater (day 6; Figure 8). By contrast, there was a significant increase in the protein abundance of Nka α 1b in the gills of these experimental fish (Figure 9). Overall, there was a significant increase in the protein abundance of Nka α-subunit, as demonstrated by the pan-specific anti-NKA (α 5) antibody, in gills of *A. testudineus* acclimated progressively from freshwater to seawater (Figure 10).

### Immunofluorescence microscopy

**Figure 11:**
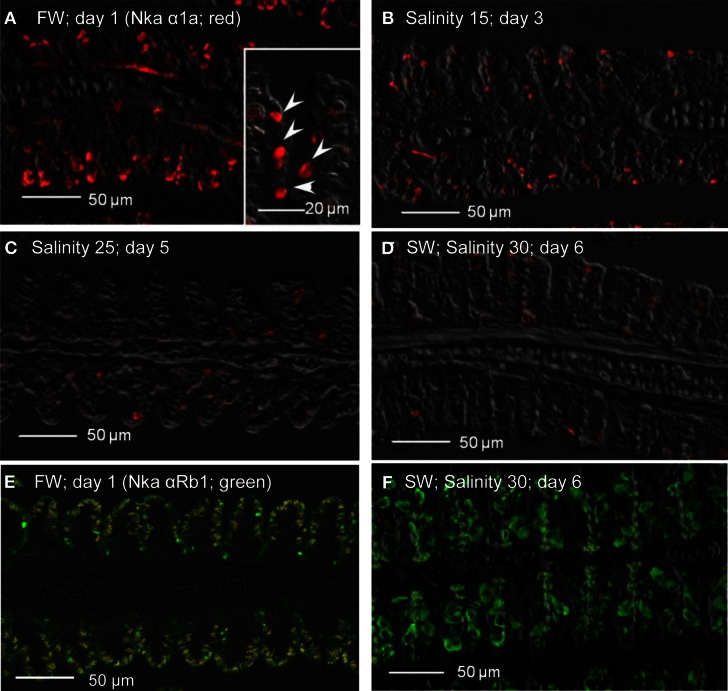
**Immunofluorescent localization of Na^+^/K^+^-ATPase α1a (Nka α1a; freshwater isoform; red) in the gills of *Anabas testudineus* kept in **(A)** freshwater on day 1 (FW; control), or after 24 h of exposure to **(B)** salinity 15 on day 3, **(C)** salinity 25 on day 5, or **(D)** salinity 30 (seawater) on day 6 during a progressive acclimation from freshwater to seawater.** Double immunofluorescence staining with anti-Nka α 1a (red) and anti-NKA α Rb1 (green) antibodies was performed on gills of fish kept in **(E)** freshwater on day 1, or **(F)** salinity 30 (seawater) on day 6. Co-localization of staining from the red and green channels resulted in a yellow-orange coloration. Sections were overlaid with the DIC images for orientation. Arrowheads in the inset of 11A indicate the staining of Nka α 1a in the freshwater-type mitochondrion-rich cells, which were smaller than the seawater-type mitochondrion-rich cells in the inset of Figure 12D, labeled by the anti-Nka α 1b antibody. Magnification: 200×; inset of **(A)**: 400×. Reproducible results were obtained from three individuals for each of the four conditions.

Immunofluorescent localization of Nka α 1a (red) revealed that it was preferentially expressed in small ovoid cells in the branchial epithelium of *A. testudineus* kept in freshwater on day 1 (Figure 11A), or exposed to salinity 15 on day 3 (Figure 11B) during a progressive acclimation from freshwater to seawater. However, expression was drastically reduced in the gills of fish exposed to salinity 25 (day 5; Figure 11C) or to seawater (day 6; Figure 11D). The Nka α Rb1 antibody (green) is known to be pan-specific for the α subunit of Nka, and indeed it co-labeled with Nka α 1a (red) in the gills of *A. testudineus* in freshwater, producing a yellow-orange color (Figure 11E). In comparison, the gills of the fish in seawater on day 6 was labeled green with Nka α Rb1 and Nka α 1a antibodies, indicating that the seawater-type MRCs did not express Nka α 1a (Figure 11F). Attempts to co-localize Nka α 1a in TUNEL positive apoptotic cells in fish exposed to salinity 25 (day 5) were unsuccessful probably because Nka α 1a would have been degraded in these cells succumbed to high salinity.

**Figure 12:**
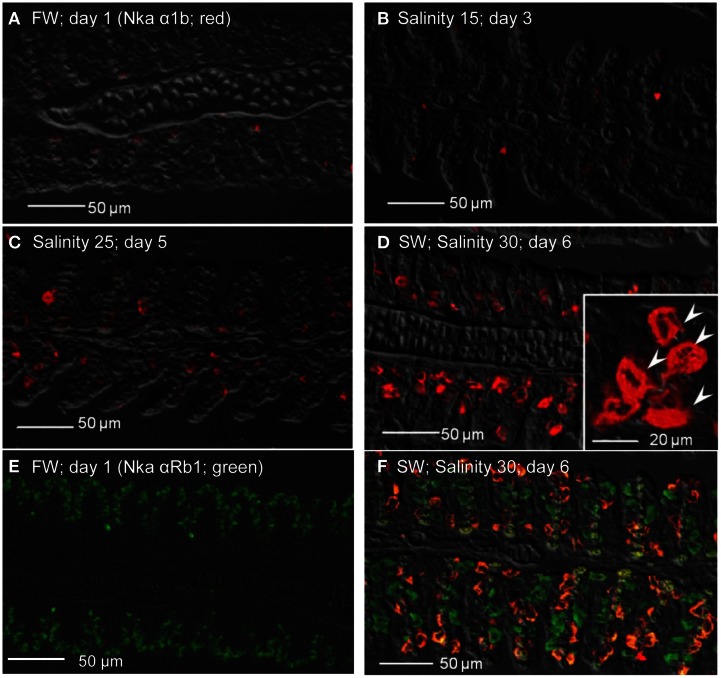
**Immunofluorescent localization of Na^+^/K^+^-ATPase α1b (Nka α1b; seawater isoform; red) in the gills of *Anabas testudineus* kept in **(A)** freshwater on day 1 (FW; control), or after 24 h of exposure to **(B)** salinity 15 on day 3, **(C)** salinity 25 on day 5, or **(D)** salinity 30 (seawater) on day 6 during a progressive acclimation from freshwater to seawater.** Double immunofluorescence staining with anti-Nka α 1b (red) and anti-NKA α Rb1 (green) antibodies was performed on gills of fish kept in **(E)** freshwater on day 1, or **(F)** salinity 30 (seawater) on day 6. Co-localization of staining from the red and green channels resulted in a yellow-orange coloration. Sections were overlaid with the DIC images for orientation. Arrowheads in the inset of 12D indicate the staining of Nka α 1b in the seawater-type mitochondrion-rich cells, which were 3–4 times larger than the freshwater-type mitochondrion-rich cells in the inset of Figure 11A, labeled by the anti-Nka α 1a antibody. Magnification: 200×; inset of **(D)**: 400×. Reproducible results were obtained from three individuals for each of the four conditions.

As for Nka α 1b (red), minimal expression was detected in the gills of fish kept in freshwater on day 1 (Figure 12A), or exposed to salinity 15 (day 3; Figure 12B), but its expression drastically increased in the gills of fish exposed to salinity 25 (day 5; Figure 12C), or to seawater (day 6; Figure 12D). In comparison, the Nka α 1b-labeled MRCs in the gills of seawater-acclimated fish were 3-4 times larger than the Nka α 1a-labeled cells in the gills of freshwater. In contrast with Nka α 1a (Figure 12E), the gills of the fish in freshwater was labeled green with Nka α Rb1 and Nka α 1b antibodies, indicating that the freshwater-type MRCs did not express Nka α 1b (Figure 12E). For fish exposed to seawater on day 6, co-labeling with Nka α Rb1 (green) and Nka α 1b (red) antibodies produced a yellow-orange color (Figure 12F).

**Figure 13:**
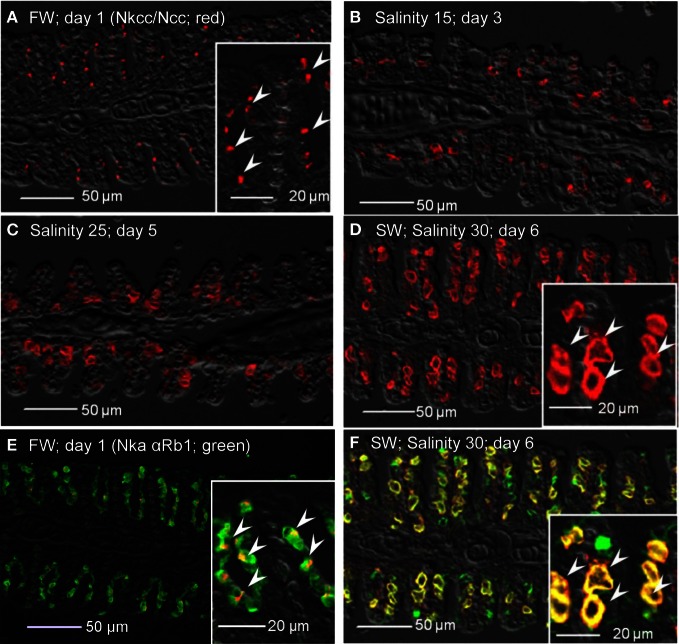
**Immunofluorescent localization of Na^+^:K^+^:2Cl^−^ cotransporter (Nkcc; red)/Na^+^:Cl^−^ cotransporter (NCC; red) in the gills of *Anabas testudineus* kept in **(A)** freshwater on day 1 (FW; control), or after 24 h of exposure to **(B)** salinity 15 on day 3, **(C)** salinity 25 on day 5, or **(D)** salinity 30 (seawater) on day 6 during a progressive acclimation from freshwater to seawater.** Double immunofluorescence staining with anti-NKCC/NCC and anti-NKA α Rb1 (green) antibodies was performed on gills of fish kept in **(E)** freshwater on day 1, or **(F)** salinity 30 (seawater) on day 6. Co-localization of staining from the red and green channels resulted in a yellow–orange coloration. Sections were overlaid with the DIC images for orientation. Arrowheads in the figure insets of panels **(A,E)** indicate the staining of Nkcc/Ncc on the apical membranes of relatively small freshwater-type mitochondrion-rich cells. Arrowheads in the figure insets of panels **(D,F)** indicate the relatively large seawater-type mitochondrion-rich cells with basolateral membrane staining by anti-NKCC/NCC and anti-NKA α Rb1 antibodies. Magnification: 200×; insets of panels **(A)**, **(D)**, **(E)**, and **(F)**: 400×. Reproducible results were obtained from three individuals for each of the four conditions.

Nkcc immune-reactivity was limited discretely to the apical membrane of the branchial epithelium of freshwater *A. testudineus* (Figure 13A). Of note, it has been established that the anti-Nkcc antibody (T4) can react not only with both basolateral Nkcc1 and apical Nkcc2 isoforms, but also with an apical Na^+^:Cl^−^ cotransporter (Ncc), which is involved in hyperosmotic regulation in fish (Hiroi et al., 2008). Therefore, it is probable that the labeling represented apical Ncc or Nkcc2 of the freshwater-type MRCs (Figure 13A), which were indeed smaller than the seawater-type MRCs (Figure 13B), corroborating the observations made with anti-Nka antibodies. Apical labeling of Ncc/Nkcc2 was apparently replaced progressively by basolateral labeling of Nkcc1 in gills of fish exposed to salinity 15 (day 3; Figure 13B) and then to salinity 25 (day 5; Figure 13C). Nkcc1 was strongly expressed in gills of fish exposed to seawater (day 6; Figure 13D). Double labeling with Nka α Rb1 (green) and Nkcc (red) antibodies indicated that basolateral Nka and apical Ncc/Nkcc2 were co-expressed in small freshwater-type MRCs in the gills of freshwater *A. testudineus* (Figure 13E). For fish exposed to seawater on day 6, Nka and Nkcc1 were co-localized (yellow-orange due to a mix of green and red) to the basolateral membrane of seawater-type MRCs (Figure 13F) which were 3–4 times larger than freshwater-type MRCs (Figure 13E).

**Figure 14:**
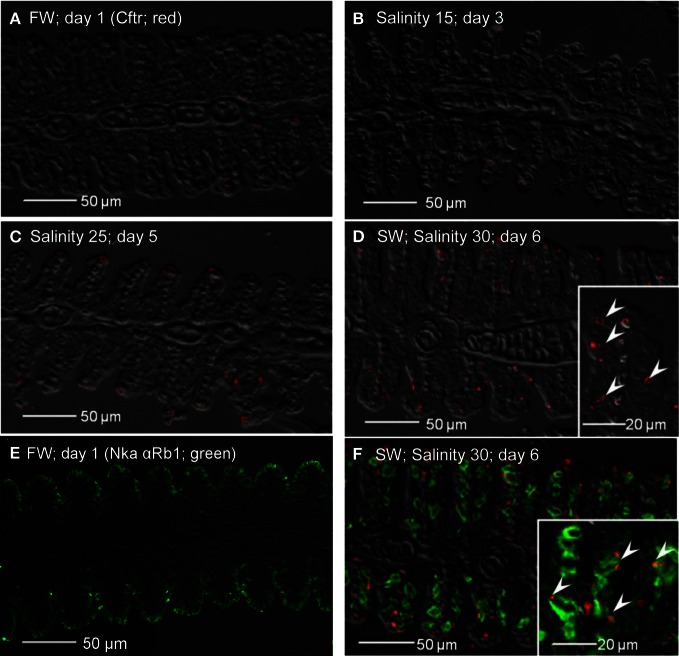
**Immunofluorescent localization of cystic fibrosis transmembrane conductance regulator (Cftr; red) in the gills of *Anabas testudineus* kept in **(A)** freshwater on day 1 (FW; control), or after 24 h of exposure to **(B)** salinity 15 on day 3, **(C)** salinity 25 on day 5, or **(D)** salinity 30 (seawater) on day 6 during a progressive acclimation from freshwater to seawater.** Double immunofluorescence staining with anti-CFTR and anti-NKA α Rb1 (green) antibodies was performed on gills of fish kept in **(E)** freshwater on day 1, or **(F)** salinity 30 (seawater) on day 6. Sections were overlaid with the DIC images for orientation. Arrowheads in figure insets indicate the staining of Cftr on the apical membranes of mitochondrion-rich cells by the anti-CFTR antibody (inset of **D**) while the basolateral membrane was labeled with the anti-NKA α Rb1 antibody (inset of **F**). Magnification: 200×; insets of **(D)** and **(F)**: 400×. Reproducible results were obtained from three individuals for each of the four conditions.

Apart from non-specific staining of red blood cells, no expression of Cftr was detected from the gills of fish kept in freshwater on day 1 (Figure 14A), or exposed to salinity 15 (day 3; Figure 14B). However, expression of Cftr in seawater-type MRCs became detectable in the gills of fish exposed to salinity 25 (day 5; Figure 14C), and was commonly observed in the gills of fish exposed to seawater (day 6; Figure 14D). Nka α Rb1 labeling (green) indicated the presence of small freshwater-type MRCs, which lacked Cftr labeling, in the gills of freshwater *A. testudineus* (Figure 14E). For fish exposed to seawater on day 6, Cftr was localized (red) to the apical membrane of large seawater-type MRCs labeled (green) with Nka αRb1 (Figure 14F).

**Figure 15:**
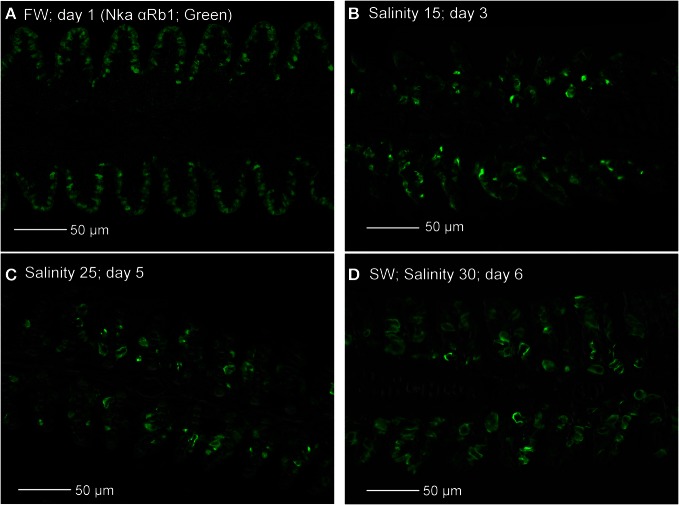
**Immunofluorescent staining of Na^+^/K^+^-ATPase (Nka; green) using the anti-NKA αRb1 antibody, for localization of mitochondrion-rich cells in the gills of *Anabas testudineus* kept in **(A)** freshwater on day 1 (FW; control), or after 24 h of exposure to **(B)** salinity 15 on day 3, **(C)** salinity 25 on day 5, or **(D)** salinity 30 (seawater) on day 6 during a progressive acclimation from freshwater to seawater.** Sections were overlaid with the DIC or brightfield images for orientation. Magnification: 400×. Reproducible results were obtained from three individuals for each of the four conditions.

Immunofluorescent staining of Nka using the anti-NKA α Rb1 antibody (Figure 15), which did not differentiate the various Nka α-isoforms, revealed that there could be a decrease in the number of functional MRCs in the gills of *A. testudineus* exposed to salinity 15 (day 3, Figure 15B) or to salinity 25 (day 5; Figure 15C) during a progressive acclimation from freshwater to seawater.

## Discussion

### Salinity-induced tunel-positive apoptosis

Apoptosis is the programmed cell death characterized by specific morphologic and biochemical properties (Kerr et al., 1972). Activation of endonucleases that cleave chromosomal DNA preferentially at inter-nucleosomal sections is one of the hallmarks of apoptosis. Hence, DNA fragmentation revealed by the presence of a multitude of DNA strand breaks is considered to be the gold standard for identification of apoptotic cells. TUNEL is a common method for detecting DNA fragmentation that results from apoptotic signaling cascades. Our results indicate for the first time that extensive TUNEL-positive apoptosis occurred in the gills of *A. testudineus* in salinity 25 (day 5) during a 6-day progressive increase in salinity from freshwater to seawater. Gills constitute a crucial respiratory organ in water-breathing fishes and therefore extensive restructuring of the branchial epithelium for osmoregulatory purposes may have undesirable effects on respiration. However, *A. testudineus* is an obligatory air-breather, which depends largely on the labyrinth organs in the upper opercular chambers for air-breathing. Perhaps because of this, it could afford extensive apoptotic cell death to facilitate anatomical/physiological restructuring of the branchial epithelium during seawater acclimation, and such a response would have little side effects on its respiration (Chang et al., 2007). However, it would imply that extensive salinity-induced apoptosis may not be a general phenomenon among gills of water-breathing euryhaline fish species during a progressive seawater acclimation.

Teleosts have evolved sophisticated iono/osmoregulatory mechanisms to inhabit freshwater and/or seawater. The main osmoregulatory organ in teleosts is the gill and the major cell type involved in ionic transport in fish gills are MRCs. MRCs of freshwater and seawater fishes are involved in ion absorption and secretion, respectively (Hwang and Lee, 2007; Evans et al., 2008; Hwang et al., 2011). During progressive seawater acclimation, there must be a change in the type of MRCs in the gills of a euryhaline freshwater fish, like *A. testudineus*. Since apoptosis is critical in the elimination of redundant, ectopic, damaged, mutated or infected cells (Santos et al., 2008), it can be concluded that branchial osmoregulatory acclimation in *A. testudineus* involved the removal, and hence also the *de novo* replacement, of certain cell types in its gills. Using electron microscopy Wendelaar Bonga et al. (1989) demonstrated that MRCs and pavement cells underwent apoptosis and replacement at a constant rate in the gills of *O. mossambicus* in freshwater. They also demonstrated that a progressive acclimation to seawater stimulated the apoptosis of MRCs, but not pavement cells. On the other hand, Inokuchi and Kaneko (2012) reported that an acute transfer of *O. mossambicus* from freshwater to brackish water (salinity 24) resulted in apoptosis of MRCs and other cell types including pavement cells. Therefore, it is logical to propose that the increase in apoptosis in the gills of *A. testudineus* exposed to salinity 25 during a progressive increase in salinity could be related to an increase in the degeneration and removal of freshwater-type MRCs, although salinity-induced apoptosis of other cell types cannot be ignored. Such a proposition was supported indirectly by the location of the apoptotic cells in the branchial epithelium (comparing Figure 1C with Figures 11A–C) and results obtained subsequently from immunofluorescence microscopy tracking the loss of the Nka freshwater-type α 1a subunit.

### Salinity-induced increase in caspase activity

Apoptotic pathways are mediated by caspases and as such, cells dying without the participation of caspases do not display the typical morphological hallmarks of apoptosis (Adrain and Martin, 2006). While the extrinsic and the intrinsic apoptotic pathways are differentially triggered and require different initiator caspases, they converge at the effector level, involving caspase-3, -6, and -7, and ultimately leading to apoptosis. Therefore, the significant increase in branchial caspase-3/-7 activity at salinity 15 (day 3; Figure 2A), which preceded the prominent TUNEL-positive apopotosis at salinity 25 (day 5; Figure 1), is in support of the proposition that increased apoptosis occurred during branchial osmoregulatory acclimation in *A. testudineus*. Because seawater acclimation led to significant increases in branchial caspase-8 and caspase-9 activities, it can be concluded that both the extrinsic and the intrinsic pathways of apoptosis had been activated, although the intrinsic pathway could predominate since the peak activity of caspase-9 was higher than that of caspase-8.

Of note, the caspase-8 (Figure 2B) and caspase-9 (Figure 2C) activities peaked at salinity 25 (day 5), but the caspase-3/-7 activity peaked at salinity 15 (day 3) instead and returned to control level at salinity 25 (day 5) (Figure 2A). Since caspase-3/-7 can be inhibited directly through interaction with inhibitors of apoptosis proteins (IAPs) or indirectly via phosphorylation of the proapoptotic protein, BAD, which participates in the intrinsic apoptosis activation cascade (Deveraux and Reed, 1999), our results indicate that some other mechanisms could have been activated to inhibit caspase-3/-7 activity from day 5 onwards, leading to a significant decrease in caspase-3/-7 activity on day 6 (1 day in SW). The inhibition of caspase-3/-7 activity could be essential to facilitate the proliferation of the seawater-type MRCs to replace the freshwater-type MRCs which had been removed through apoptosis. Why would caspase-8 and -9 activities be maintained high when caspase-3/7 activities had already returned to control level on day 5? Several studies have demonstrated an essential role for caspase-8 in the proliferation of immune cells (Chun et al., 2002; Salmena et al., 2003; Beisner et al., 2005; Su et al., 2005). Caspase-8 is also required for the myelomonocytic lineage differentiation into macrophages (Kang et al., 2004). Whether sustaining up-regulation of caspase-8 into salinity 25 (day 5) had a functional role in increased differentiation and proliferation of branchial epithelial cells, especially seawater-type MRCs, in the gills of *A. testudineus* during seawater acclimation is unclear at present.

### Salinity-induced increase in *p53*/p53 expression

p53 can transcriptionally and non-transcriptionally regulate the membrane expression of the death receptors, and p53-mediated apoptosis can proceed through death receptor signaling (the extrinsic pathway) and activation of effector caspases (see Schuler and Green, 2001 for a review). While death receptor signaling and alternative pathways may contribute to the full development of p53-mediated apoptosis, the intrinsic apoptotic pathway is both necessary and sufficient for stress-induced and p53-dependent caspase activation, (Schuler and Green, 2001). Here, we report for the first time increases in *p53* mRNA expression and p53 protein abundance in the gills of a teleostean fish in response to seawater acclimation. While mammals regulate p53 activity through post-translational modification with little induction in p53 mRNA levels (Kastan et al., 1991), regulation of p53 activity in fish can also involve transcription (Lee et al., 2008). Indeed, a significant increase in *p53* mRNA expression and a progressive increase in p53 protein abundance were observed in *A. testudineus* exposed to salinity 10 (day 2) and salinity 20 (day 4), respectively, indicating that the signal for apoptosis began early during a progressive acclimation from freshwater to seawater. Of note, the plasma of teleostean fishes are generally isoosmotic to water of salinity 10, which implies that they would have to begin hypoosmotic/hypoionic osmoregulation from salinity 10 onwards. After reaching a peak in seawater on day 6, the mRNA expression of *p53* returned to control level after 6 days of acclimation at seawater (day 11), implying the completion of the apoptotic modifications needed for branchial osmoregulatory acclimation.

### Salinity-induced increase in *bax* expression

The intrinsic pathway of apoptosis is regulated by the Bcl-2 family proteins, including BAX, and characterized by an increase in the mitochondrial membrane permeability to cytochrome C (Green and Reed, 1998; Kroemer et al., 2007). In mammals, the release of mitochondrial cytochrome C can be triggered through p53-induced activation of BAX in a caspase-independent manner, and cells from mice deficient in caspase 9 or Apaf-1 were resistant to DNA damage-induced apoptosis and to p53-dependent death stimuli (see Schuler and Green, 2001 for a review). Zebrafish Bax can substitute for mammalian BAX in BAX/BAK-deficient cells (Valentijn et al., 2008) in spite of its limited N-terminus sequence homology to mammalian proteins. In addition, similar to *BAX* in mammals, *bax* in zebrafish embryo is transcriptionally upregulated together with *cyclin G*1, *mdm2* and *p*21^*Waf*1/*Cip*−1^ when treated with p53-activating agents (Lee et al., 2008). The fact that seawater acclimation led to a significant increase in the mRNA expression of *bax* in the gills of *A. testudineus*, is in support of the proposition that salinity-induced apoptosis could have occurred, in part, through the activation of the intrinsic pathway and related caspases. While a significant increase in *p53* mRNA expression occurred at salinity 10 (day 2), a 1.7-fold increase in *bax* mRNA expression was observed later at salinity 20 (day 4). Since p53 acts as a transcriptional activator of *bax*, it would take time for the increased *p53* transcript to be reflected as an increase in p53 activity, and for the increased p53 activity to be translated into an upregulation of *bax*. As in the case of *p53*, the mRNA expression of *bax* returned to the control level in the gills of *A. testudineus* after 6 days of acclimation in seawater (day 11).

### Transition between freshwater-type and seawater-type MRCs, and its time course relationship with increased apoptosis

Increases in Nka activity upon exposure to a hyperosmotic environment can be due to an increase in *nka* α-subunit mRNA expression leading to an increase in Nka protein abundance (Lee et al., 2000; Singer et al., 2002; Tipsmark et al., 2002; Lin et al., 2003, 2004, 2006; Scott et al., 2004) and/or a modulation of the enzyme's hydrolytic rate (Crombie et al., 1996; Bystriansky et al., 2007). Changes in mRNA expression of *nka* α-subunit isoforms during seawater acclimation have been examined in gills of *Onchorhynchus mykiss*, *Salvelinus alpinus*, *Salmo salar* (Richards et al., 2003; Bystriansky et al., 2006, 2007; Nilsen et al., 2007), *Onchorhynchus nerka* (Shrimpton et al., 2005), *Fundulus heteroclitus* (Scott et al., 2004) and *A. testudineus* (Ip et al., 2012a). Ip et al. (2012a) obtained results which suggested that *nka* α*1a* was a freshwater isoform involved in ion absorption and *nka* α*1b* was a seawater isoform essential for ion secretion in a hyperosmotic environment in *A. testudineus*. In this study, we developed and validated by Western blotting two monoclonal antibodies raised specifically against the freshwater Nka α1a and seawater Nka α1b isoforms of *A. testudineus*. Through using these antibodies in immunofluorescence microscopy, we demonstrated for the first time the presence of distinct freshwater-type and seawater-type Nka-specific MRCs in the gills of *A. testudineus*. More importantly, we confirmed that the expression of Nka α 1a was reduced during and after TUNEL-positive apoptosis had occurred in salinity 25 water. These results provide indirect support to the proposition that branchial osmoregulatory acclimation in *A. testudineus* could involve the degeneration and the subsequent removal of some, if not all, freshwater-type MRCs.

Immunofluorescence microscopy revealed drastic increases in expression of Nka α 1b, Nkcc1 and Cftr in a distinctly larger type of MRC after the occurrence of TUNEL-positive apoptosis in the gills of *A. testudineus*. Although labeling with T4 anti-Nkcc/Ncc antibody indicated the possible appearance of basolateral Nkcc in the gills of fish exposed to salinity 15 on day 3, labeling with Nka α 1b antibody suggested that the seawater-type MRCs did not appear until exposure to salinity 25 on day 5. Taken altogether, our results indicate indirectly that freshwater-type MRCs could be removed and replaced by seawater-type MRCs in the gills of *A. testudineus* during seawater acclimation. That could have contributed to the decrease in number of functional MRCs in the gills of *A*. exposed to salinity 15 on day 3 or to salinity 25 on day 5. However, the percentage of freshwater-type MRCs undergoing apopotosis and the cellular origin of the seawater-type MRCs are uncertain at present. Of note, Wong and Chan (1999) used flow cytometry to study isolated branchial cells of the Japanese eel, *Anguilla japonica*, and identified several types and subtypes of MRCs, based on cell size, cellular granularity and cell autofluorescence. They proposed a population of non-MRCs with high mitotic activity as stem cells in the gills of *A. japonica* (Wong and Chan, 1999). Recently, the presence of epithelial stem cells has been confirmed in zebra fish (Hsiao et al., 2007; Jänicke et al., 2007; Horng et al., 2009; Conte, 2012). Therefore, the possibility that undifferentiated cells or stem cells were involved in the generation of seawater-type MRCs in the gills of *A. testudineus* during a progressive acclimation from freshwater to seawater could not be eliminated.

## Conclusion

Extensive apoptosis in the branchial epithelium of *A. testudineus* occurred in salinity 25 (day 5) during a 6-day progressive acclimation from freshwater to seawater, as evident by increased positive TUNEL staining and caspase activity, and upregulation of *p53*/p53 and *bax* expression. These results indicate that branchial osmoregulatory acclimation in *A. testudineus* involved the removal of certain types of branchial cell through apoptosis. Immunoflourescence microscopy using specific anti-Nka α-isoform, anti-Nkcc and anti-Cftr antibodies confirmed the presence of freshwater-type MRCs and seawater-type MRCs in the gills of *A. testudineus* exposed to freshwater and seawater, respectively. Since modulation of existing MRCs during seawater acclimation could not have led to extensive salinity-induced apoptosis, our results indicate that branchial osmoregulatory acclimation in *A. testudineus* might involve the removal of freshwater-type MRCs through apoptosis and their subsequent replacement by seawater-type MRCs. Such a proposition is supported indirectly by the time course events between the peak of increased apoptosis and the transition between freshwater-type MRCs and seawater-type MRCs in the gills of *A. testudineus* during a progressive acclimation from freshwater to seawater. However, based on our results, the possibility of some freshwater-type MRCs being able to survive the progressive increase in salinity and transform to seawater-type MRCs cannot be eliminated, and therefore warrants future studies.

### Conflict of interest statement

The authors declare that the research was conducted in the absence of any commercial or financial relationships that could be construed as a potential conflict of interest.
